# Supplementary material for: Isolation of PCV3 from Perinatal and Reproductive Cases of PCV3-Associated Disease and In Vivo Characterization of PCV3 Replication in CD/CD Growing Pigs
Source: Viruses. 2020 Feb 16;12(2):219. doi: 10.3390/v12020219 (PMC7077311; doi:10.3390/v12020219)
Supplement: Supplementary file 1 [file viruses-12-00219-s001.pdf]

Figure S1.

A

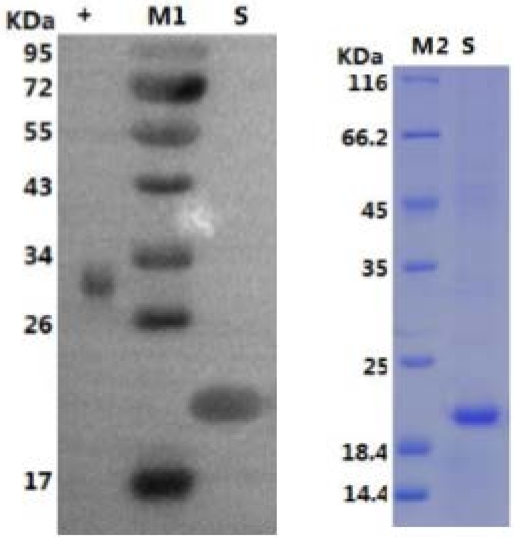

B

PCV3 ORF2 protein sequence:
MNHKVHHHHHHMSVGTPQNNKPWHANHFITRLNEWETAITFEYYKILKMKVTLSPVIS
PAQQTKTMFGHTAIDLDGAWTTNTWLQDDPYAESSTRKVMTSKKKHSRYFTPKPLLAG
TTSAHPGQSLFFFSRPTPWLNTYDPTVQWGALLWSIYVPEKTGMTDFYGTKEVWIRYKS VL

Figure S1. Purification of PCV3 recombinant ORF2 protein. **(A)** SDS-PAGE and Western Blot results of the purified PCV3 ORF2 recombinant protein. M1: pre-stained protein ladder protein marker; M2: protein marker; S: purified recombinant PCV3 ORF2 protein (0.3 mg/ml in 20mM Tris, 300 mM NaCl, 0.5% SKL, pH 8.0); +: positive control. **(B)** PCV3 ORF2 protein sequence.

Figure S2.

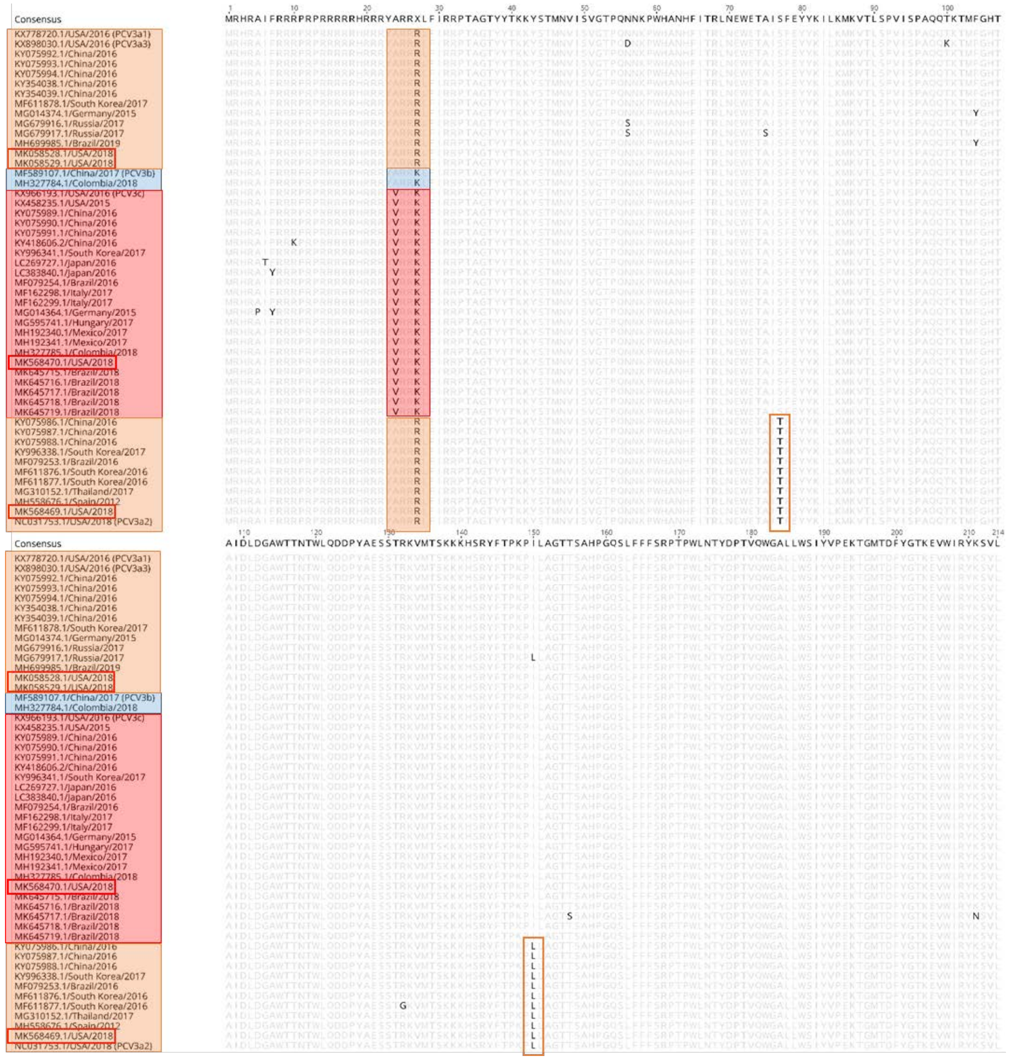

Figure S2. Molecular features of the four PCV3 isolates and full-length Cap (ORF2) protein alignment. Four full-length PCV3 cap protein from three PCV3 isolates (red boxes) and 46 reference strains aligned by Geneious R9 software. Two amino acid mutations are used for PCV3 clade classification (A24 V and R27 K). Strain name and amino acid mutation are highlighted in orange, blue and red for PCV3a, PCV3b and PCV3c respectively. Additional subclade classification within clade PCV3a was based on mutation of amino acids S77T, and I150L. Amino acid changes (S77T and I105L) highlighted in orange boxes shows strain classified within subclade PCV3a2.
